# Mediating Effects of Global Negative Effect Expectancies on the Association between Problematic Cannabis Use and Social Anxiety

**DOI:** 10.3389/fpsyt.2017.00249

**Published:** 2017-11-22

**Authors:** Maria Di Blasi, Paola Cavani, Laura Pavia, Crispino Tosto, Sabina La Grutta, Rosa Lo Baido, Cecilia Giordano, Adriano Schimmenti

**Affiliations:** ^1^Department of Psychological and Educational Sciences, University of Palermo, Palermo, Italy; ^2^Department of Experimental Biomedicine and Clinical Neuroscience, University of Palermo, Palermo, Italy; ^3^Department of Human and Social Sciences, UKE, Kore University of Enna, Enna, Italy

**Keywords:** social anxiety, cannabis use, expectancies, young adults, substance use

## Abstract

The relationship between social anxiety (SA) and cannabis use among adolescents and young adults is a highly debated topic. In this cross-sectional study, we tested whether cannabis use expectancies mediated the association between SA and cannabis use severity in a sample of 343 young adults (74.3% male) who used cannabis. They completed self-report measures for the screening of problematic cannabis use (Cannabis Use Problems Identification Test) and SA symptoms (Social Interaction Anxiety Scale and Social Phobia Scale). A multiple mediation analysis was used to test whether marijuana effect expectancies mediate SA effect on problematic cannabis use. SA was negatively associated with cannabis use severity in this sample, and we found evidence that cannabis use expectancies fully mediated this relationship. Specifically, global negative effect expectancies influence the relationship between SA and problematic cannabis use. These findings may inform current prevention strategies and clinical intervention for young adults who use cannabis.

## Introduction

Social anxiety is the most common form of psychological suffering among adolescent and youth with an early age of onset (by age 11 years in about 50% and by age 20 years in about 80% of individuals (1)), and subsequent negative developmental outcomes (2). Several studies in both adolescent and adult samples have well documented the comorbidity of social anxiety (SA) with other disorders, and its negative consequences on psychological functioning (3–6). Extensive literature has also analyzed the relationship between SA and substance use, although results remain unconclusive (7).

Among other illicit drugs, cannabis use has become very common in adolescents and youth, with growing evidence of negative health consequences and increased risk for dependence and difficulties with school for early heavy users (8). Additionally, there is substantial research linking SA symptoms with cannabis use in adolescence and young adulthood. Several cross-sectional and longitudinal studies among adolescents have found a negative association between SA and cannabis use, suggesting that SA might play a protective role against cannabis involvement (9–12). A possible explanation for the protective effect of SA is that, due to their avoidant behaviors, socially anxious adolescents are less likely to be involved in peer groups in which cannabis use is frequent. Moreover, their apprehensive traits could hinder the involvement with cannabis use to avoid the fear of embarrassing themselves when intoxicated. Differently, previous studies among young adults found that high levels of SA were related, even perspectively, to cannabis use disorder (abuse or dependence), and several use-related problems (13–16). According to the self-medication hypothesis (17), it has been suggested that high-social anxious youth may use cannabis to reduce emotional distress and to cope with unpleasant social situations.

Expectancies regarding the effects of substance use have proved to be a promising area in explaining the link between SA disorder and substance use. According to social learning models of addiction, expectancies are defined as beliefs and attitudes regarding the effects of a given substance and represent an important construct to explain initiation, maintenance, and cessation of substance consumption (18–20). Positive attitudes or expectancies concerning substance or alcohol use during adolescence and youth seem to be predictive of current and subsequent substance use or problematic involvement (21, 22). Specifically, cannabis use expectancies change among users and non-users and in relation to different patterns of use (20, 23, 24). Specifically, beliefs about the undesirable effects of cannabis and negative expectancies are stronger among non-users and play a protective role against the involvement in more frequent and problematic use; moreover, among consumers, positive expectancies become more salient to, and predictive of, frequency of use, dependence severity, and relapse (25, 26). However, some studies found that negative expectancies were more associated to cannabis use and related problems than the positive ones (27–29).

To date, literature investigating the role of expectancies in explaining the relationship between SA and cannabis use is still sparse. Buckner and Schmidt (28) found in a sample of undergraduate students that among individuals with higher levels of SA, marijuana users reported significantly greater cognitive, and behavioral impairment expectancies than non-users. Consistently, another study conducted on undergraduate users (30) found that individuals with SA disorder had more cognitive and behavioral impairment (CBI) and global negative effects (GNE) expectancies compared with those without the diagnosis of SA and that these expectancies mediated the relationship between SA and marijuana-related problems. A third study (31) provided similar findings showing that patients with SA disorder who were users or had been dependent on cannabis reported stronger cognitive impairment and negative effects expectancies when compared with individuals without cannabis use and psychiatric disorders. Differently, two recent studies of community adolescent samples reported a negative association between cannabis use and SA, and, respectively, found that cognitive impairment and negative behavioral effects (12) and social and sexual facilitation (SSF) expectancies acted as mediators of this inverse relationship (10).

The present study sought to evaluate the mediating role of cannabis use expectancies on the relationship between SA and cannabis use in a community sample of undergraduate students. Specifically, the aims of the study were twofold: (a) to assess the relationship between SA and cannabis use and (b) to analyze whether specific cannabis effect expectancies mediate this relationship. On the basis of previous findings (28, 30), we hypothesized that higher levels of SA were associated with increasing involvement in problematic cannabis use. Moreover, in line with prior work (28) we hypothesized that, among lower-order expectancies, CBI, and global negative expectancies would mediate the link between SA and problematic marijuana use.

## Materials and Methods

### Participants and Procedure

This cross-sectional study originally involved 671 college students (69.4% female; *n* = 466), recruited on the basis of voluntary participation, from 17 college courses of three Universities located in three different cities of a Southern region of Italy (Sicily). Eligibility criteria were: being 18–30 years old, and self-reported no current substance abuse or psychiatric treatment. All the participants were Caucasian and mean age was 23.23 years (SD = 2.55, range = 18–30). Data for the present study were obtained from 343 participants who were current cannabis consumers and completed the Cannabis Use Problems Identification Test (CUPIT) (32) to assess the degree of involvement in cannabis use and the Social Interaction Anxiety Scale and the Social Phobia Scale (SIAS and SPS) to assess SA. Participants in the current study include those that completed these self-report measures. The group was mainly composed of male participants (74.3%) and ranged in age from 19 to 28 years old (M = 23.32, SD = 2.52). Heads’ Departments approval for aims’ study and all research procedures were obtained prior to students’ participation in the study. Within each university students were recruited from the first and the fourth years of courses of a wide variety of academic disciplines (Humanities, Education, Medicine, Biology, Physical fitness, Psychology, Jurisprudence, Engineering, Architecture, and Economics).

After obtaining written informed consent, all measures were group administrated by two trained researchers at the beginning or at the end of the courses’ lessons. Participants were informed of the study’s aims and measures and told they could omit any information they did not wish to provide and could withdraw from the study at any time. A confidential identification code was created for each participant and was used for all identifying information. Data were gathered from October to June 2015. No participant refused participation. The study was conducted in accordance with the Declaration of Helsinki and with the ethical guidelines for psychological research laid down by the Italian Psychological Association.

### Measures

#### Cannabis Use

The CUPIT (32) is a self-report for the detection of potentially problematic cannabis use. The total CUPIT score is obtained by summing items raw scores. Possible scores range from 1 (non-problematic use) to 82 (severely/dependent problematic use). Total scores ranging from 1 to 11 identify non-problematic users, from 12 to 19 risky users, and from 20 to 82 problematic users. CUPIT demonstrates high-internal consistency and test–retest reliability across diverse community settings and consumers of all ages (32). In the present sample, the total CUPIT score was used as a measure of problematic cannabis use. The measure demonstrated adequate internal consistency in this sample (α = 0.80).

#### Marijuana Expectancies

Expectancies were assessed using the 48-item Marijuana Effect Expectancies Questionnaire (MEEQ) (19). The MEEQ is comprised of six lower-order scales and two higher-order scales. The overall scale has good psychometric properties, including good reliability, convergent, and discriminant validity (23). In this study, only the lower-order scales were considered, which demonstrated adequate internal consistency in the sample: CBI (α = 0.81), relaxation and tension reduction (α = 0.84), SSF (α = 0.68), perceptual and cognitive enhancement (α = 0.73), GNE (α = 0.80), and craving and physical effects (CPE) (α = 0.68).

#### Social Anxiety

Social anxiety was assessed with the SIAS and SPS (33) according to Sica et al. (34) criteria of the Italian adaptation. The SIAS and SPS are self-report companion measures commonly used in the SA literature (35). They assess two different domains of SA: social interaction anxiety in dyads or groups, and fears of being observed by others, respectively. The SIAS comprises 19 items (range scores 0–76, cut-off = 25) and SPS consists of 20 items (range scores 0–80, cut-off = 19) both rated on a five-point Likert-type scale. The measures demonstrated excellent levels of internal consistency and test–retest reliability across several samples (36–39). Higher scores indicate greater SA symptoms. In the present sample, the internal consistencies of SIAS and SPS were α = 0.88 and 0.90, respectively. Since SIAS and SPS are companion measures (33), their scores were mean-centered and combined in order to obtain a total score of SA (40).

Cannabis Use Problems Identification Test and MEEQ were translated according to guidelines that are widely accepted for the translation of instruments in cross-cultural research (41). The back translation was reviewed by the author of each instrument. Differences in the original and the back-translated versions were discussed and resolved by joint agreement of translators.

### Data Analysis

**Figure 1:**
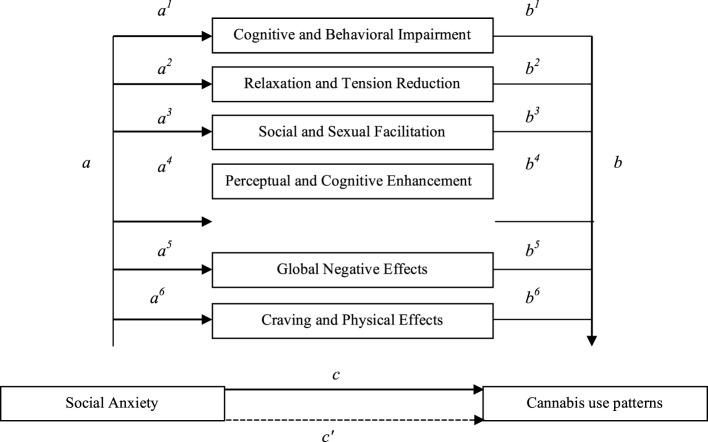
Theoretical mediation model of the relationship between social anxiety (SA) and cannabis use patterns *via* cannabis effect expectancies. Note *a*: predictor on mediators (effects of SA on marijuana effect expectancies); *b*: mediators on outcome (effects of marijuana effect expectancies on cannabis use patterns); *c*: predictor on outcome without mediators (effect of SA on cannabis use pattern); *c*′: predictor on outcome with mediators (direct effect of SA on cannabis use pattern controlling for marijuana effect expectancies); *ab*: indirect effects of predictor on outcome *via* mediators (indirect effects of SA on cannabis use pattern *via* marijuana effect expectancies).

First, descriptive statistics were calculated for all the study variables. Subsequently, linearity was assessed through the visual inspection of bivariate scatterplots for all possible dependent variable pairings, as recommended by Tabachnick and Fidell (42). Because the assumption of linearity appeared to hold reasonably well, we computed Pearson’s bivariate correlations between SA, marijuana effect expectancies, and cannabis use problems. To test whether marijuana effect expectancies mediate SA effect on cannabis use patterns, we tested a multiple mediation through the Model 4 of Preacher and Hayes’ multiple mediation SPSS computational tool (43, 44), controlling for socio-demographic variables (gender and age). Figure 1 shows the theoretical mediation model; a total effect (*c*) refers to the relationship between CUPIT and SA without controlling for Marijuana Effect Expectancies. A direct effect (*c*′) refers to the relationship between CUPIT and SA after controlling for Marijuana Effect Expectancies. A total indirect effect (*ab*) refers to the role of all Marijuana Effect Expectancies in the relationship between CUPIT and SA. A specific indirect effect (*a*^1^*b*^1^, *a*^2^*b*^2^, *a*^3^*b*^3^, *a*^4^*b*^4^, *a*^5^*b*^5^, and *a*^6^*b*^6^) refers to the role of a specific Marijuana Effect Expectancy in the relationship between CUPIT and SA.

To prevent violation of normal distribution assumption, a non-parametric bootstrapping method with 5,000 bootstrap samples was used as a robust estimation of both direct and indirect effects (43). Bootstrapping provided a confidence interval (CI) around the indirect effect of the independent variable (SA) on the dependent variable (CUPIT) *via* the mediators (MEEQ scales). Multiple regressions are significant if the interval between the lower limit and the upper limit of a bootstrapped 95% CI do not contain zero, which means that the mediating effect is different from zero. In this study, the mediating model for the 95% CI of the indirect path was obtained using 5,000 resamples.

## Results

Means, SDs, and intercorrelations between SA, CUPIT, and MEEQ scales were calculated (Table 1). In the current study, CUPIT scores ranged from 1 to 64 (M = 4.88, SD = 8.68). Most of the participants reported a non-problematic cannabis use (74.3%), and 92 (26.8%) and 40 (11.7%) participants were risky and problematic users, respectively. SA showed a negative correlation with CUPIT (*r* = −0.79, *p* < 0.01) and positive correlation with all marijuana effect expectancies scales, except for CBI and CPE.

**Table 1:** Means, SDs, and intercorrelations of measures.

Measures	1	2	3	4	5	6	7	8
1. SA	–	–0.079*	0.055	0.081*	0.124**	0.130**	0.141**	0.006
2. CUPIT		–	−0.057	0.049	−0.014	−0.040	−0.407**	0.263**
3. CBI			–	0.376*	0.283**	0.438**	0.639**	0.567**
4. RTR				–	0.692**	0.741**	0.283**	0.500**
5. SSF					–	0.754**	0.344**	0.418**
6. PCE						–	0.458**	0.486**
7. GNE							–	0.269**
8. CPE								–
Mean	0	4.88	33.75	25.44	26.74	23.43	25.62	20.33
SD	1	8.68	7.58	7.25	6.23	6.20	7.16	4.68

*SA, social anxiety; CUPIT, Cannabis Use Problems Identification Test; CBI, cognitive and behavioral impairment; RTR, relaxation and tension reduction; SSF, social and sexual facilitation; PCE, perceptual and cognitive enhancement; GNE, global negative effects; CPE, craving and physical effects*.

*p < 0.05.

**p < 0.001.

First, we tested whether the six marijuana effect expectancies (MEEQ scales) mediated the relationship between SA and CUPIT (Table 2). Socio-demographic variables were not associated with the mediators (age, *p* = 0.887 ranging from −0.15 to 0.129, ns; gender, *p* = 0.01 ranging from −1.79 to −0.244, ns). Only one expectancy appeared to fully mediate the link between the two variables. Specifically, it was found that SA was negatively associated with CUPIT [*B* = −0.959, 95% CI = −0.17 to 0.04, *t*_(343)_ = −2.32, *p* = 0.020] and positively related to GNE expectancy [*B* = 0.806, 95% CI = 0.14 to 1.46, *t*_(343)_ = 2.737, *p* = 0.01]. Lastly, results indicated that the mediator GNE was negatively associated with CUPIT [*B* = −0.689, 95% CI = −0.79 to −0.58, *t*_(343)_ = −12.435, *p* = 0.000]. Results showed the mediating role of GNE in the relationship between SA and CUPIT [*B* = −0.555; 95% CI = −1.06 to −0.08, *t*_(343)_ = −2.249, *p* = 0.02]. In addition, the results indicated that the direct effect of SA on CUPIT became non-significant [*B* = −0.170, 95% CI = −0.085 to 0.51, *t*_(343)_ = −0.488, *p* = 0.625] when controlling for GNE, thus suggesting full mediation.

**Table 2:** Summary of multiple mediation analysis of SA and CUPIT (5,000 bootstraps).

Independent variable	Mediators	Dependent variable	Effect of IV on M	Effect of M on DV	Direct effect	Indirect effect		Total effect of IV on DV

IV	M	DV	(*a*)	(*b*)	(*c*′)	(*a* × *b*)	95% CI	c
					−0.170 (SE = 0.349)			−0.959 (SE = 0.41)*
SA	CBI	CUPIT	0.03 (SE = 0.37)	0.11 (SE = 0.05)*		0.003	(−0.096; 0.125)	
	RTR		0.48 (SE = 0.34)	−0.02 (SE = 0.06)		−0.014	(−0.165; 0.047)	
	SSF		0.85 (SE = 0.29)*	0.002 (SE = −0.07)		0.002	(−0.134; 0.145)	
	PCE		0.77 (SE = 0.29)*	0.02 (SE = 0.08)		0.022	(−0.109; 0.214)	
	GNE		0.80 (SE = 0.33)*	−0.68 (SE = 0.05)**		−0.555*	(−1.062; −0.085)	
	CPE		−0.21 (SE = 0.22)	0.67 (SE = 0.08)**		−0.147	(−0.515; 0.139)	

*SA, social anxiety; CUPIT, Cannabis Use Problems Identification Test; CBI, cognitive and behavioral impairment; RTR, relaxation and tension reduction; SSF, social and sexual facilitation; PCE, perceptual and cognitive enhancement; GNE, global negative effects; CPE, craving and physical effects*.

*p < 0.05.

**p < 0.001.

## Discussion

The present study aimed to examine the relationship between SA and problematic cannabis use and to evaluate the mediating role of marijuana effects expectancies in this relationship. With regard to the first aim, contrary to the hypothesis, SA was found to be negatively related to cannabis use. Previous studies involving adolescent samples (9–11, 45, 46) showed that SA was a negative predictor of the risk of cannabis use and could therefore play a protective function. Although current findings may be to some extent limited by the cross-sectional nature of the study, is therefore likely that socially anxious youth, similar to adolescents, may use less cannabis because they are less likely to find themselves in peer context where cannabis use is common, probably due to their avoidant behavior. However, these findings challenge those of other studies (15, 30), that found a positive relationship between SA and cannabis use problems among similar samples. Several reasons could account for this discrepancy. First, methodological differences could contribute since different measures of SA use have been used. Earlier studies of Buckner et al. relied on a structured diagnostic interview to detect mood and anxiety disorders (30) and on a single measure of SA assessing fear and avoidance of social situations (15). On the contrary, in this study two companion measures were used to assess the different domains of SA: social interaction anxiety, and fear of being scrutinized in specific performance situations (e.g., eating, drinking, formal speaking, and in the presence of others). Although these methodological differences make it difficult to compare our results with those of Buckner et al., it could be possible to argue that, in the present study, the detection of fear of negative evaluation by others may in part explain the negative association between SA and problematic cannabis use. In fact, it is possible that those who have an intense fear of negative evaluations by others in performance situations are more likely to be particularly concerned about using cannabis due to its altering effects on mind and behavior.

Moreover, in this study current users were mainly non-problematic while other studies sampled populations of more experienced and heavier user. As a consequence, it can be argued that reasons for cannabis consumption may be different in light versus heavy consumers. Second, cultural and environmental factors such as cannabis availability might help to explain the difference between our findings and those of previous studies. Although several studies have shown that the availability seems to be a relevant influencing factor of initiation and progression to symptoms of cannabis abuse (47, 48), literature about the relationship between availability, law on cannabis possession, and cannabis use is yet far from conclusive (49, 50). It is beyond the scope of this article reviewing laws on cannabis use, but it is important to note that the Italian law system does not differentiate cannabis-related offenses (such as possession for personal use) from those of any other illegal drugs. For this reason, it is possible to hypothesize that due to the apprehensive traits characterizing high-socially anxious youth, Italian youth could be significantly afraid to transgress social norms and avoid situations and contexts related to the purchasing of illegal substances.

On the other hand, a body of literature has also tried to assess whether or not cannabis use may increase the risk for developing anxiety disorder. Evidence for the predictive role of cannabis consumption on the onset of anxiety disorders is still weak. Recently, two meta-analysis of longitudinal studies (51, 52) on the association between cannabis use and anxiety symptoms in the general population concluded that cannabis use is a minor risk factor for the development of elevated anxiety symptoms in the general population. With specific regard to the onset of SA disorder, a recent longitudinal study (53) assessing the bidirectional association between cannabis use and anxiety disorders in a population-based adult sample found a trend linking problematic cannabis use with the future development of SA. More specifically, cannabis use disorder was predictive of subsequent higher incidence of SA among adults aged 18–29. Despite the cross-sectional nature of the present study prevents us from making any causal inference, our result of a negative association between involvement in cannabis use and levels of SA does not appear to support the findings of the cited study. Given the paucity of research evaluating the association between cannabis use and subsequent onset of SA symptoms and disorders, future longitudinal studies are needed to better understand the nature of these relationships, especially among young adults.

The second aim of this study was to analyze whether marijuana effect expectancies mediate the relationship between problematic cannabis use and SA. In the present study, the inverse relationship between SA and problematic cannabis consumption is totally attributable to the mediating role of GNE expectancies. Consistent with previous research, results from this study provide support to the notion that youth who perceive undesired or unpleasant effects of cannabis consumption are less likely engaged in problematic cannabis use. In fact, a large body of cross-sectional and longitudinal research (20, 22, 54, 55) found a significant negative relationship between negative cannabis use expectancies and the frequency of cannabis use, thus confirming that the endorsement of negative use expectancies may protect from involvement in cannabis use. Moreover, it could be hypothesized that negative effects expectancies about the potential effects of cannabis consumption may further reinforce the high levels of fearful traits characterizing socially anxious youth. On the other hand, our results do not support previous research in this area (28, 30) that found that global negative expectancies mediated the positive relationship between SA and increasing marijuana-related problems. We rather found that global negative expectancies fully mediated the negative association between SA and problematic involvement in cannabis consumption. The difference between our findings and those of Buckner and Schmidt (28, 30) could be attributed to the same differences mentioned above. Specifically, they may be in part due to the different measures of SA that have been used and to differences in sample involvement in heavy cannabis use. However, a possible explanation for our data is that global negative expectancies on marijuana consumption represent substance effects that seem particularly undesirable for socially anxious individuals. The global negative expectancies subscale is composed of items such as “Marijuana makes me say things I do not mean,” “Marijuana causes me to lose control and became careless,” and “Marijuana makes me critical and short-tempered.” As suggested by items content, social anxious young adults may be less likely to be attracted by the effects of cannabis because of specific individual characteristics related to the disorder (e.g., heightened self-consciousness, social fears, and discomfort in social interactions) thus providing a convincing explanation for the relationship between higher symptoms of SA and less problematic involvement in cannabis consumption found in the present study.

The current study has two main strengths. First of all, differently from previous studies, it used a relatively large sample of cannabis users attending different faculties and specialization derived from three universities. Second, the study contributes to the sparse existing literature providing a novel and unexpected insights into the explaining role of expectancies in the relationship between SA and cannabis use. In spite of these strengths, some limitations need to be highlighted which can in turn provide directions for future research. First, due to the cross-sectional design, conclusions about processes over time or causal relationships between variables cannot be drawn. Although the direction of the relationships among SA, cannabis use expectancies, and cannabis use patterns are invoked based on theory and are supported by relevant literature, a reversal causation cannot be ruled out due to the cross-sectional nature of the study. In fact, it is possible that cannabis use expectancies have direct and indirect effects on anxiety states that can alter the cannabis use patterns. As a consequence, future longitudinal works are needed to better evaluate the relationship between SA, expectancies about cannabis consumption, and problematic cannabis use, also controlling for potential confounders (56). Second, since the sample was comprised of a heterogeneous community sample of college students, further research using clinical samples is required. Moreover, in addition to the variables tested in the present study, future studies should consider their interaction with other factors, such personality traits close to SA (e.g., shyness, introversion) and psychiatric disorders such as major depressive disorder panic disorder, posttraumatic stress disorder that are the most frequent principal diagnosis in patients with comorbid SAD (5, 57). Socio-economics factors could also play a potential role since recent literature indicate that during the last decade recession hardships increase psychological distress (58, 59) which in turn increases marijuana and other illicit drugs use (60). Additionally, as recent studies highlighted that high-potency and synthetic cannabinoids compared with the use of natural cannabis, may cause more frequent and more severe unwanted negative effects such as agitation, paranoia, psychosis (61, 62), further studies should be conducted to consider the influence of type of cannabis smoked on the SA symptoms. Finally, because it is possible that the law system pertaining to the legal status of cannabis use may influence the relationship between SA and habits of consummation, studies comparing countries with different law system should be undertaken.

## Conclusion

The increasing prevalence rate of cannabis consumption and the recent debate on the legal status of cannabis use suggest a careful examination of risk and protective factors for problematic cannabis use. The present study adds to the current body of knowledge on this issue showing that SA symptoms have a negative association with problematic cannabis involvement among young adults. Specifically, the findings show that the negative relationship between SA and problematic cannabis use is fully mediated by the presence of negative expectations about the effects of cannabis consumption. The result could have important implications for the prevention of problematic cannabis use. As SA symptoms show a substantial link with negative social and mental health outcomes, it cannot be considered and strategically promoted as a resilience factor against problematic involvement in cannabis use among young people. Nevertheless, our findings indicate that preventive strategies focused on maintaining negative expectancies about the effects of cannabis could increase a potential protective role of SA among socially anxious young adults. Moreover, since previous studies (15, 30) have shown that higher SA worsens the cannabis-related problems among heavy user students, a preventive screening for SA symptoms in the academic setting might help to intercept the need for more tailored intervention for high-socially anxious students in order to prevent the negative consequences related to heavy cannabis use in this population.

## Ethics Statement

This study was carried out in accordance with the recommendations of the Ethical Code of the University of Palermo and of the Code of Ethics approved by the General Assembly of the Italian Association of Psychology held on March 27, 2015. All subjects gave written informed consent in accordance with the Declaration of Helsinki. Ethical approval was not required for this study in accordance with the national and institutional guidelines.

## Author Contributions

MB, PC, LP, and CT designed the study and managed the research process. PC, LP, and CT collected and analyzed the data. MB performed the literature review and took primary responsibility for initial drafting. All authors agree to be accountable for all aspects of the work in ensuring that questions related to the accuracy or integrity of any part of the work are appropriately investigated and resolved. SG, RB, CG, and AS gave important supervision for drafting the final version of the manuscript.

## Conflict of Interest Statement

The authors declare that the research was conducted in the absence of any commercial or financial relationships that could be construed as a potential conflict of interest.

## References

[1] Stein MB Stein DJ. Social anxiety disorder. Lancet (2008) 371(9618):1115–25. 10.1016/S0140-6736(08)60488-2 18374843

[2] Merikangas KR He JP Burstein M Swanson SA Avenevoli S Cui L Lifetime prevalence of mental disorders in US adolescents: results from the National Comorbidity Survey replication—adolescent supplement (NCS-A). J Am Acad Child Adolesc Psychiatr (2010) 49(10):980–9. 10.1016/j.jaac.2010.05.017 PMC2946114 20855043

[3] Di Blasi M Cavani P Pavia L Lo Baido R La Grutta S Schimmenti A The relationship between self-image and social anxiety in adolescence. Child Adolesc Ment Health (2015) 20(2):74–80. 10.1111/camh.12071 32680392

[4] Fehm L Beesdo K Jacobi F Fiedler A. Social anxiety disorder above and below the diagnostic threshold: prevalence, comorbidity and impairment in the general population. Soc Psychiatry Psychiatr Epidemiol (2008) 43(4):257–65. 10.1007/s00127-007-0299-4 18084686

[5] Lydiard RB. Social anxiety disorder: comorbidity and its implications. J Clin Psychiatry (2001) 62(Suppl 1):17–24. 11206030

[6] Xu Y Schneier F Heimberg RG Princisvalle K Liebowitz MR Wang S Gender differences in social anxiety disorder: results from the national epidemiologic sample on alcohol and related conditions. J Anxiety Disord (2012) 26(1):12–9. 10.1016/j.janxdis.2011.08.006 21903358

[7] Buckner JD Heimberg RG Ecker AH Vinci C. A biopsychosocial model of social anxiety and substance use. Depress Anxiety (2013) 30(3):276–84. 10.1002/da.22032 23239365

[8] Coffey C Patton GC. Cannabis use in adolescence and young adulthood: a review of findings from the Victorian Adolescent Health cohort study. Can J Psychiatry (2016) 61(6):318–27. 10.1177/0706743716645289 27254840 PMC4872246

[9] Fröjd S Ranta K Kaltiala-Heino R Marttunen M Association of social phobia and general anxiety with alcohol and drug use in a community sample of adolescents. Alcohol Alcohol (2011) 46(2):192–9. 10.1093/alcalc/agq096 21245062

[10] Di Blasi M Pavia L Cavani P Lo Verso G Schimmenti A Cannabis use and social anxiety in adolescence: the role of facilitation expectancies. J Child Adolesc Subst Abuse (2015) 24(6):397–404. 10.1080/1067828X.2013.872066

[11] Schmits E Mathys C Quertemont E. A longitudinal study of cannabis use initiation among high school students: effects of social anxiety, expectancies, peers and alcohol. J Adolesc (2015) 41:43–52. 10.1016/j.adolescence.2015.02.009 25800726

[12] Schmits E Mathys C Quertemont E Is social anxiety associated with cannabis use? The role of cannabis use effect expectancies in middle adolescence. J Child Adolesc Subst Abuse (2016) 25(4):348–59. 10.1080/1067828X.2015.1039683

[13] Buckner JD Schmidt NB Bobadilla L Taylor J. Social anxiety and problematic cannabis use: evaluating the moderating role of stress reactivity and perceived coping. Behav Res Ther (2006) 44(7):1007–15. 10.1016/j.brat.2005.08.002 16168950

[14] Buckner JD Schmidt NB Lang AR Small JW Schlauch RC Lewinsohn PM. Specificity of social anxiety disorder as a risk factor for alcohol and cannabis dependence. J Psychiatr Res (2008) 42(3):230–9. 10.1016/j.jpsychires.2007.01.002 17320907 PMC2254175

[15] Buckner JD Heimberg RG Schmidt NB. Social anxiety and marijuana-related problems: the role of social avoidance. Addict Behav (2011) 36(1):129–32. 10.1016/j.addbeh.2010.08.015 20832947 PMC2981690

[16] Buckner JD Heimberg RG Schneier FR Liu SM Wang S Blanco C. The relationship between cannabis use disorders and social anxiety disorder in the National Epidemiological Study of Alcohol and Related Conditions (NESARC). Drug Alcohol Depend (2012) 124(1):128–34. 10.1016/j.drugalcdep.2011.12.023 22266089 PMC3350824

[17] Khantzian EJ. The self-medication hypothesis of substance use disorders: a reconsideration and recent applications. Harv Rev Psychiatry (1997) 4(5):231–44. 10.3109/10673229709030550 9385000

[18] Marlatt GA Gordon JR Relapse Prevention: Maintenance Strategies in the Treatment of Addictive Behaviours. New York: Guilford Press (1985).

[19] Schafer J Brown SA. Marijuana and cocaine effect expectancies and drug use patterns. J Consult Clin Psychol (1991) 59(4):558–65. 10.1037/0022-006X.59.4.558 1918560

[20] Brackenbury LM Ladd BO Anderson KG Marijuana use/cessation expectancies and marijuana use in college students. Am J Drug Alcohol Abuse (2016) 42(1):25–31. 10.3109/00952990.2015.1105242 26678375 PMC4767562

[21] Stone AL Becker LG Huber AM Catalano RF. Review of risk and protective factors of substance use and problem use in emerging adulthood. Addict Behav (2012) 37(7):747–75. 10.1016/j.addbeh.2012.02.014 22445418

[22] Schmits E Maurage P Thirion R Quertemont E. Dissociation between implicit and explicit expectancies of cannabis use in adolescence. Psychiatry Res (2015) 230(3):783–91. 10.1016/j.psychres.2015.11.005 26575651

[23] Aarons GA Brown SA Stice E Coe MT. Psychometric evaluation of the marijuana and stimulant effect expectancy questionnaires for adolescents. Addict Behav (2001) 26(2):219–36. 10.1016/S0306-4603(00)00103-9 11316378

[24] Alfonso J Dunn ME. Differences in the marijuana expectancies of adolescents in relation to marijuana use. Subst Use Misuse (2007) 42(6):1009–25. 10.1080/10826080701212386 17613960

[25] Hayaki J Hagerty CE Herman DS de Dios MA Anderson BJ Stein MD. Expectancies and marijuana use frequency and severity among young females. Addict Behav (2010) 35(11):995–1000. 10.1016/j.addbeh.2010.06.017 20621423 PMC2919625

[26] Boden MT McKay JR Long WR Bonn-Miller MO. The effects of cannabis use expectancies on self-initiated cannabis cessation. Addiction (2013) 108(9):1649–57. 10.1111/add.12233 23627879 PMC6440474

[27] Simons JS Arens AM. Moderating effects of sensitivity to punishment and sensitivity to reward on associations between marijuana effect expectancies and use. Psychol Addict Behav (2007) 21(3):409–14. 10.1037/0893-164X.21.3.409 17874892

[28] Buckner JD Schmidt NB. Marijuana effect expectancies: relations to social anxiety and marijuana use problems. Addict Behav (2008) 33(11):1477–83. 10.1016/j.addbeh.2008.06.017 18694625 PMC2556243

[29] Foster DW Jeffries ER Zvolensky MJ Buckner JD. The interactive influence of cannabis-related negative expectancies and coping motives on cannabis use behavior and problems. Subst Use Misuse (2016) 51(11):1504–11. 10.1080/10826084.2016.1188947 27356272 PMC4965297

[30] Buckner JD Schmidt NB. Social anxiety disorder and marijuana use problems: the mediating role of marijuana effect expectancies. Depress Anxiety (2009) 26(9):864–70. 10.1002/da.20567 19373871 PMC2773507

[31] Guillem E Notides C Vorspan F Debray M Nieto I Leroux M Cannabis expectancies in substance misusers: French validation of the marijuana effect expectancy questionnaire. Am J Addict (2011) 20(6):543–54. 10.1111/j.1521-0391.2011.00171.x 21999501

[32] Bashford J Flett R Copeland J. The cannabis use problems identification test (CUPIT): development, reliability, concurrent and predictive validity among adolescents and adults. Addiction (2010) 105(4):615–25. 10.1111/j.1360-0443.2009.02859.x 20403014

[33] Mattick RP Clarke JC Development and validation of measures of social phobia scrutiny fear and social interaction anxiety. Behav Res Ther (1998) 36(4):455–70. 10.1016/S0005-7967(97)10031-6 9670605

[34] Sica C Musoni I Chiri LR Bisi B Lolli V Sighinolfi C Social phobia scale e social interaction anxiety scale: traduzione e adattamento italiano. Bollettino di Psicologia Applicata (2007) 252:59–71.

[35] McNeil DW Ries BJ Turk CL Behavioral assessment: self-report, physiology, and overt behavior. In: Heimberg RG Liebowitz MR Hope DA Schneier FR, editors. Social Phobia: Diagnosis, Assessment, and Treatment. New York: Guilford (1995). p. 202–31.

[36] Brown EJ Turovsky J Heimberg RG Juster HR Brown TA Barlow DH Validation of the social interaction anxiety scale and the social phobia scale across the anxiety disorders. Psychol Assess (1997) 9(1):21–7. 10.1037/1040-3590.9.1.21

[37] Carleton RN Collimore KC Asmundson GJ McCabe RE Rowa K Antony MM. Refining and validating the social interaction anxiety scale and the social phobia scale. Depress Anxiety (2009) 26(2):E71–81. 10.1002/da.20480 19152346

[38] Hedman E Ljótsson B Rück C Furmark T Carlbring P Lindefors N Internet administration of self-report measures commonly used in research on social anxiety disorder: a psychometric evaluation. Comput Human Behav (2010) 26(4):736–40. 10.1016/j.chb.2010.01.010

[39] Kupper N Denollet J. Social anxiety in the general population: introducing abbreviated versions of SIAS and SPS. J Affect Disord (2012) 136(1):90–8. 10.1016/j.jad.2011.08.014 21903277

[40] Ham LS Hope DA. Incorporating social anxiety into a model of college problem drinking: replication and extension. Psychol Addict Behav (2006) 20(3):348–55. 10.1037/0893-164X.20.3.348 16938075 PMC2652650

[41] Brislin RW Back translation for cross-cultural research. J Cross Cult Psychol (1970) 1(3):185–216. 10.1177/135910457000100301

[42] Tabachnick BG Fidell LS Using Multivariate Statistics. 5th ed New York: Allyn and Bacon (2007). 980 p.

[43] Preacher KJ Hayes AF. Asymptotic and resampling strategies for assessing and comparing indirect effects in multiple mediator models. Behav Res Methods (2008) 40(3):879–91. 10.3758/BRM.40.3.879 18697684

[44] Hayes AF Preacher KJ Statistical mediation analysis with a multicategorical independent variable. Br J Math Stat Psychol (2013) 67(3):451–70. 10.1111/bmsp.12028 24188158

[45] Myers MG Aarons GA Tomlinson K Stein MB. Social anxiety, negative affectivity, and substance use among high school students. Psychol Addict Behav (2003) 17(4):277–83. 10.1037/0893-164X.17.4.277 14640823

[46] Nelemans SA Hale WW Raaijmakers QA Branje SJ van Lier PA Meeus WH. Longitudinal associations between social anxiety symptoms and cannabis use throughout adolescence: the role of peer involvement. Eur Child Adolesc Psychiatry (2016) 25(5):483–92. 10.1007/s00787-015-0747-8 26254219 PMC4854944

[47] Gillespie NA Neale MC Kendler KS Pathways to cannabis abuse: a multi-stage model from cannabis availability, cannabis initiation and progression to abuse. Addiction (2009) 104(3):430–8. 10.1111/j.1360-0443.2008.02456.x 19207351 PMC2844887

[48] Franelic P Kuzman M Simetin IP Kern J. Impact of environmental factors on marijuana use in 11 European countries. Croat Med J (2011) 52(4):446–57. 10.3325/cmj.2011.52.446 21853539 PMC3160692

[49] Turnbull PJ. The great cannabis classification debacle: what are the likely consequences for policing cannabis possession offences in England and Wales? Drug Alcohol Rev (2009) 28(2):202–9. 10.1111/j.1465-3362.2008.00045.x 19320707

[50] Bestrashniy J Winters KC. Variability in medical marijuana laws in the United States. Psychol Addict Behav (2015) 29(3):639–42. 10.1037/adb0000111 26415061 PMC4588056

[51] Twomey CD. Association of cannabis use with the development of elevated anxiety symptoms in the general population: a meta-analysis. J Epidemiol Community Health (2017) 71(8):811–6. 10.1136/jech-2016-208145 28053188

[52] Kedzior KK Laeber LT A positive association between anxiety disorders and cannabis use or cannabis use disorders in the general population-a meta-analysis of 31 studies. BMC Psychiatry (2014) 14(1):136 10.1186/1471-244X-14-136 24884989 PMC4032500

[53] Feingold D Weiser M Rehm J Lev-Ran S The association between cannabis use and anxiety disorders: results from a population-based representative sample. Eur Neuropsychopharmacol (2016) 26(3):493–505. 10.1016/j.euroneuro.2015.12.037 26775742

[54] Pedersen ER Miles JN Osilla KC Ewing BA Hunter SB D’Amico EJ. The effects of mental health symptoms and marijuana expectancies on marijuana use and consequences among at-risk adolescents. J Drug Issues (2015) 45(2):151–65. 10.1177/0022042614559843 25977590 PMC4428682

[55] Kristjansson SD Agrawal A Lynskey MT Chassin LA. Marijuana expectancies and relationships with adolescent and adult marijuana use. Drug Alcohol Depend (2012) 126(0):102–10. 10.1016/j.drugalcdep.2012.04.024 22682980 PMC3798067

[56] Valeri L VanderWeele TJ Mediation analysis allowing for exposure–mediator interactions and causal interpretation: theoretical assumptions and implementation with SAS and SPSS macros. Psychol Methods (2013) 18(2):137 10.1037/a0035596 23379553 PMC3659198

[57] Dalrymple KL Zimmerman M. Does comorbid social anxiety disorder impact the clinical presentation of principal major depressive disorder? J Affect Disord (2007) 100(1):241–7. 10.1016/j.jad.2006.10.014 17188365 PMC2547849

[58] Van Hal G. The true cost of the economic crisis on psychological well-being: a review. Psychol Res Behav Manag (2015) 8:17–25. 10.2147/PRBM.S44732 25657601 PMC4295900

[59] Di Blasi M Tosto C Marfia A Cavani P Giordano C Transition to adulthood and recession: a qualitative study. J Youth Stud (2016) 19(8):1043–60. 10.1080/13676261.2015.1136055

[60] Nagelhout GE Hummel K de Goeij MC de Vries H Kaner E Lemmens P. How economic recessions and unemployment affect illegal drug use: a systematic realist literature review. Int J Drug Policy (2017) 44:69–83. 10.1016/j.drugpo.2017.03.013 28454010

[61] Bassir Nia A Medrano B Perkel C Galynker I Hurd YL. Psychiatric comorbidity associated with synthetic cannabinoid use compared to cannabis. J Psychopharmacol (2016) 30(12):1321–30. 10.1177/0269881116658990 27462088

[62] Fattore L Synthetic cannabinoids—further evidence supporting the relationship between cannabinoids and psychosis. Biol Psychiatry (2016) 79(7):539–48. 10.1016/j.biopsych.2016.02.001 26970364

